# Spatial and temporal variation in prey color patterns for background matching across a continuous heterogeneous environment

**DOI:** 10.1002/ece3.6024

**Published:** 2020-02-22

**Authors:** Marleen Baling, Devi Stuart‐Fox, Dianne H. Brunton, James Dale

**Affiliations:** ^1^ Unitec Institute of Technology Auckland New Zealand; ^2^ School of Natural and Computational Sciences Massey University (Albany Campus) Auckland New Zealand; ^3^ School of BioSciences The University of Melbourne Melbourne VIC Australia

**Keywords:** camouflage, crypsis, intraspecific signaling, temporal variation

## Abstract

In heterogeneous habitats, camouflage via background matching can be challenging because visual characteristics can vary dramatically across small spatial scales. Additionally, temporal variation in signaling functions of coloration can affect crypsis, especially when animals use coloration seasonally for intraspecific signaling (e.g., mate selection). We currently have a poor understanding of how wild prey optimize background matching within continuously heterogeneous habitats, and whether this is affected by requirements of intraspecific signaling across biological seasons. Here, we quantified color patterns of a wild population of shore skink (*Oligosoma smithi*), a variably colored lizard endemic to New Zealand, to (a) investigate whether background matching varies across a vegetation gradient; (b) assess potential signaling functions of color; and (c) to determine whether there is a trade‐off between requirements for crypsis and intraspecific signaling in coloration across seasons. Although all pattern types occurred throughout the vegetation gradient, we found evidence for background matching in skinks across the vegetation gradient, where dorsal brightness and pattern complexity corresponded with the proportion of vegetation cover. There was also a significant disparity between ventral color (saturation) of juveniles and adults, and also between sexes, suggestive of sex recognition. However, there was little indication that color was condition‐dependent in adults. Despite some evidence for a potential role in signaling, crypsis did not greatly differ across seasons. Our study suggests that selection favors a mix of generalist and specialist background matching strategies across continuously heterogeneous habitats.

## Graphical abstract

There is currently a poor understanding of how wild prey optimize background matching within continuously heterogeneous habitats, and whether this is affected by requirements of intraspecific signaling across biological seasons. Our study on a population of variably colored lizard species showed their background matching corresponded with the gradient of vegetation cover at the site. Crypsis in the population did not differ across seasons despite some evidence for a role of color in intraspecific signaling. Our study suggests that selection favors a mixed background matching strategy across continuously heterogeneous habitats.

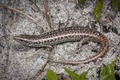

## 1 INTRODUCTION

Optimizing camouflage through background matching can be challenging for populations living in heterogeneous habitats, where visual characteristics of the background can vary across space and time. When visual characteristics of habitats vary across small spatial scales, background matching can either be optimized for a specific habitat type or represent a compromise for several habitats (Houston, Stevens, & Cuthill, 2007; Merilaita, Lyytinen, & Mappes, 2001; Merilaita, Tuomi, & Jormalainen, 1999). However, temporal variation can also affect selection for crypsis, especially when animals use coloration for intraspecific signaling only during certain times of the year (e.g., breeding vs. nonbreeding seasons). Currently, we have a poor understanding of how wild prey optimize background matching within continuously heterogeneous habitats, and whether this is affected by requirements of intraspecific signaling across biological seasons.

When habitat color, structure, and complexity vary across space, mobility of individuals within these habitat types can exert a strong influence on the extent and distribution of color patterns within that population. For example, when individuals are primarily restricted to a single background type either because of small home range or if habitat characteristics are too disparate from one another (e.g., brown vs. green backgrounds), the population is expected to undergo disruptive selection for distinctive color pattern types (e.g., brown vs. green morphs; Dale, 2006; Houston et al., 2007; Merilaita et al., 2001; Merilaita et al., 1999; Nilsson & Ripa, 2010). In such conditions, animals can achieve optimal background matching only in one background type (specialist strategy), resulting in a discrete spatial distribution of color pattern variants across the site. In contrast, populations where individuals can range over two or more background types (e.g., sparse vs. dense vegetation) can exhibit color patterns that may more generally match the different background characteristics. Despite being an inaccurate match to the entire occupied range, a compromise background matching strategy (generalists) is expected to minimize predation risk within the population (Houston et al., 2007; Hughes, Liggins, & Stevens, 2019; Merilaita et al., 2001, 1999).

Many studies on background matching have looked at disjunct habitat patches (Hughes et al., 2019; Merilaita, 2003; Merilaita & Dimitrova, 2014; Merilaita et al., 2001, 1999), but few have quantified variation in background matching along a continuum of a habitat's visual characteristics. In particular, it is unclear how the need for background matching affects the color patterns of a population living in an area that varies continuously from one extreme habitat (e.g., open area) to another extreme (e.g., highly vegetated area).

In addition to spatial differences within a habitat, temporal variation in the functional benefits for coloration can affect background matching. First, predation risk itself can vary over time due to changes in the composition of the predator species, their population density, or their foraging activity, which consequently, can alter the selection on prey color patterns for background matching (Bond, 2007; Caro, Sherratt, & Stevens, 2016; Endler, 1978). Second, seasonal changes in habitat structure and color (e.g., white snow in winter and green vegetation in summer) can also affect the selection of phenotypic characteristics for crypsis (Mills et al., 2013; Rojas, 2016; Steen, Erikstad, & Høidal, 1992; Tullberg, Gamberale‐Stille, Bohlin, & Merilaita, 2008; Zimova, Mills, Lukacs, & Mitchell, 2014). Finally, color patterns can have other biological functions that may periodically vary, such as the need for intraspecific signaling during the breeding season. Because conspecific interactions such as aggression, territoriality, and mate attraction often involve conspicuous coloration, these functions can increase the risk of being detected by predators. This balance between potentially antagonistic selection for crypsis and conspicuous signaling may affect the degree of background matching if dorsal coloration varies seasonally. Alternatively, seasonal variation in coloration for signaling may be restricted to ventral body regions hidden from visual predators (Stuart‐Fox, Moussalli, Johnston, & Owens, 2004; Stuart‐Fox & Ord, 2004). However, few studies have assessed temporal variation in both background matching and color signals.

Here, we quantified color patterns of a highly variable lizard species across space and seasons. Our study had three objectives: (a) to investigate the spatial variation in color patterns for background matching across a vegetation gradient; (b) to assess the potential signaling function of color variation by testing for correlations with age, sex, and quality; and (c) to determine if there is a trade‐off between the need for crypsis and intraspecific signaling in color patterns across seasons along a vegetation gradient. For the first objective, we predicted that dorsal color matching and pattern matching to the background would correlate with the vegetation gradient because of selection for effective crypsis. For the second objective, we expected that color patterns that are used for intraspecific signaling could be identified by color pattern differences between age (breeding adult vs. juveniles), within sexes, or color pattern corresponding to body condition or size (as a signal of individual quality). For the third objective, we predicted that if color pattern for intraspecific signaling varies across seasons, the degree of dorsal background matching would be lower during the breeding season compared with the nonbreeding season. Alternately, background matching may not be significantly affected if coloration for intraspecific signaling is only on ventral regions.

## 2 MATERIALS AND METHODS

### 2.1 Study species and study site

**Figure 1:**
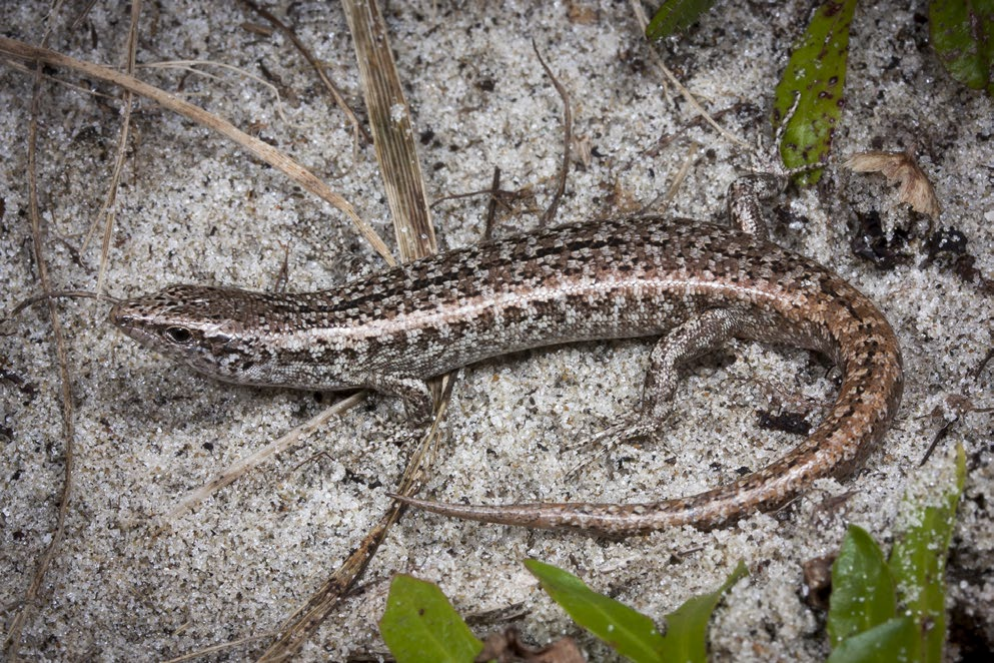
Shore skink (*Oligosoma smithi*) on the mainland North Island, New Zealand

The shore skink (*Oligosoma smithi*) is a small (adult snout–vent length, SVL = 50–70 mm) diurnal species that is endemic to New Zealand (Hitchmough et al., 2016) (Figure 1). This species exhibits among the greatest variation in color patterns for New Zealand species (both between and within populations), ranging from pale white, gray, various shades of patterned brown to uniform black (Hardy, 1977). It is not known if the color patterns of shore skinks are genetically determined. Shore skinks inhabit the coastline on the mainland and offshore islands off the North Island, New Zealand (Baling, Stuart‐Fox, Brunton, & Dale, 2016; Towns, Neilson, & Whitaker, 2002). They occupy various coastal habitats from the high tide mark to >1 km inland (ranging from dunes, vegetation scrub, rocky boulders to pebble beach) (Towns, 1975). Their home range and dispersal behavior are unknown. Both sexes exhibit aggressive behavior toward conspecifics (M. Baling, pers. obs.); however, it is unknown if this aggression is linked to territoriality because this species can aggregate in the wild. For example, >50 individuals were found in vegetation under an old plywood sheet (c. 1 × 0.7 m) (M. Baling, pers. obs.) at our study site.

We studied shore skinks at Tāwharanui Regional Park (Tāwharanui), a 550 ha mainland open sanctuary located at a peninsula within the Hauraki Gulf of Auckland, New Zealand (Maitland, 2011). The park is protected by a predator‐proof fence where all introduced mammals were eradicated in 2004, except for house mice (*Mus musculus*), European rabbits (*Oryctolagus cuniculus*), and European hedgehogs (*Erinaceus europaeus*). There are predatory birds in the park, both introduced (e.g., Australian magpie *Cracticus tibicen*, common myna *Acridotheres tristis*) and native (e.g., pukeko *Porphyrio melanotus*, sacred kingfisher *Todiramphus sanctus*). The coastal sand dune at Tāwharanui is north‐facing and consisted of pale sand and a vegetation gradient, from no or low proportion of vegetation cover in the foreshore to 100% vegetation cover at the back of the dunes (Wedding, 2007). The shore skink population at our study site had highly variable dorsal body color and patterns, and early observations suggested differences in coloration among body regions (i.e., paler ventral coloration; Baling, 2017).

### 2.2 Population surveys

We monitored the shore skink population at Tāwharanui every three months between November 2006 and May 2008. We used three existing permanent pitfall trap grids that covered the full width of the area, from the foreshore to the back of the dunes (Wedding, 2007). These grids were positioned toward one end of the beach and furthest away from the public. Two grids were spaced 75 m, and the third was 120 m apart following the coastline. One grid measured 75 × 180 m, and the other two were 100 × 140 m, totaling 4.15 ha of the area surveyed. All grids spanned the full width of the dune, from the foreshore to back of the dunes (i.e., 120–180 m). Each grid contained 40 pitfall traps, with each trap placed every 20 × 25 m (i.e., 120 traps for all three grids). Pitfall traps are 4‐L plastic buckets flushed to the ground and covered by a wooden lid with a small gap between the bucket and the lid. We used fish‐based cat food as bait, which was replaced at each check. We checked traps every 24 hr for three trap‐nights per survey. All individuals were marked on the lateral region with a xylene‐free pen before release. This was to avoid the same individuals being resampled during the same survey session.

For each captured skink, we used an Olympus Mju 770SW (Olympus, Japan) to take three photographs: dorsal side, ventral side, and the habitat background (1 × 1 m) where the skink was caught. We included a photographic gray standard (QPcard 101, Sweden) with 18% reflectance within each photograph and saved the photographs as standardized digital JPG‐file. We also recorded the sex, body mass, and length (snout to vent length, SVL) of each skink.

### 2.3 Quantifying patterns and habitat from photographs

**Figure 2:**
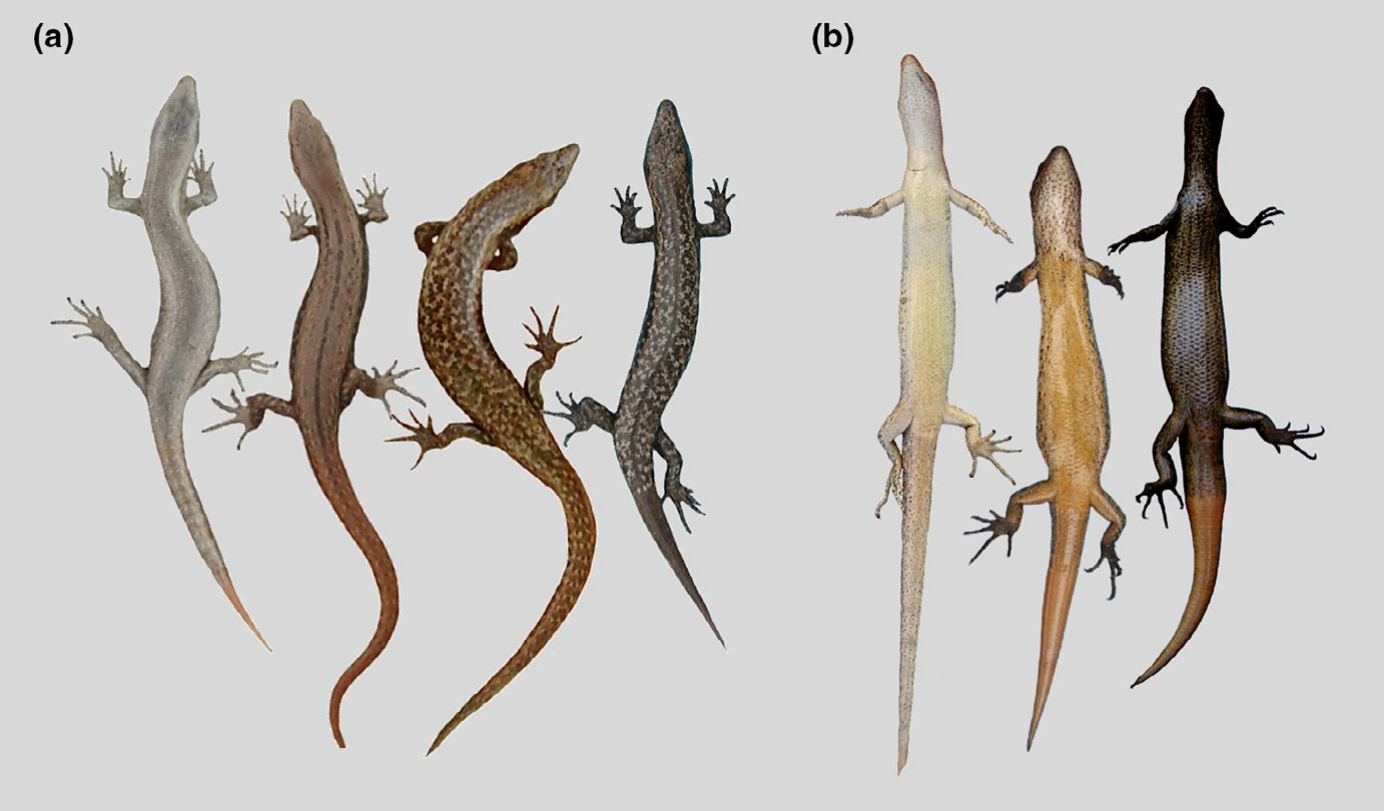
Range of body color patterns of the shore skink population at Tāwharanui Regional Park: (a) four dorsal body pattern types, from left to right; *plain*, *midplain*, *spot*, and *midspot*; (b) Examples of ventral colors, ranging from both extreme ends of their coloration, with an intermediate in the middle

We scored skinks according to the degree of their dorsal pattern complexity, as described in Baling et al. (2016). We assigned individuals to one of four pattern types: (a) *plain*: no patterns or weakly patterned; (b) *midplain*: no or weak speckling combined with the presence of a mid‐dorsal line on more than 50% of the body length; (c) *spot*: distinctive dense speckling and no (or <50%) mid‐dorsal line on length of body; and (d) *midspot*: distinctive dense speckling and presence of mid‐dorsal line in more than 50% of the body length (Figure 2). For the assessments of habitats, we estimated the amount of vegetation in the photographs. We quantified habitat as the proportion of vegetation cover, whereby each habitat photograph was divided into a 4 × 4 grid, and the proportion was estimated by eye (minimum 5%, maximum 25% in each grid section). We then summed the four section scores to produce a final score out of 100%.

### 2.4 Quantifying color from photographs

Our study species shows minimal ultraviolet (UV, 300–400 nm) reflectance (see Baling et al., 2016). Therefore, we used photographs rather than a spectrophotometer (Endler, 1990; Montgomerie, 2006) to quantify the colors because digital photography can provide information on variations for both color and patterns. Additionally, the distribution of lizard colors within RGB (see below) color space was shown to be highly correlated with the distribution of colors in avian or lizard visual color space for similarly colored lizards (Smith et al., 2016). For each photograph, we used the R package colorZapper version 1 (Dale, Dey, Delhey, Kempenaers, & Valcu, 2015; Valcu & Dale, 2014) to extract the mean values of the red (R), green (G), blue (B), and brightness (V) from 400 random points within selected areas of the gray standard, three areas of the body (dorsal: body; ventral: body and base of ventral tail), and habitat background. For background, we selected two polygon areas similar to the size of the skinks within each photograph, extracted values as above, and averaged the values of the two areas. We then calibrated the colors using linearization and equalization protocols according to Baling et al. (2016). This method standardizes color in digital photographs using measured reflectance values (Stevens, Párraga, Cuthill, Partridge, & Troscianko, 2007). We estimated saturation as the distance from the origin, and hue as the angle relative to the axis of a 2‐dimensional color space derived from RGB values (Endler, 1990). This entailed first calculating the standardized differences between the calibrated R and G channels as, *x* = (R − G)/(R + G+B), and between G and B channels as, *y *= (G − B)/(R + G+B). We then calculated saturation (S) as:

$$S={\left(x^{2}+y^{2}\right)}^{\frac{1}{2}},$$

and hue (H) as:

$$H=\tan^{-1}\left(\frac{y}{x}\right),$$

where *x* and *y* represent the standardized difference of R‐G and G‐B channels, respectively.

### 2.5 Calculating color contrasts (background matching)

For an index of background matching, we calculated the degree of achromatic and chromatic contrast between each body area (dorsal, ventral, and ventral tail) and backgrounds using the calibrated RGB values (Cadena, Smith, Endler, & Stuart‐Fox, 2017). Achromatic contrast was calculated as relative differences in brightness between the skink and its background:

$$\text{achromatic contrast}=\frac{\left(R_{s}+G_{s}+B_{s}\right)-\left(R_{b}+G_{b}+B_{b}\right)}{\left(R_{s}+G_{s}+B_{s}\right)+\left(R_{b}+G_{b}+B_{b}\right)},$$

where subscripts *s* and *b* represent skink and background, respectively.

We estimated chromatic contrast by calculating the Euclidean distance of each body area and its respective background using the proportions of each of the calibrated values of RGB (e.g., R/(R + G+B)), where the sum of R, G, and B equals to 1 (R + G+B = 1)

$$\text{chromatic contrast}=\sqrt{{\left(R_{\mathrm{ps}}-R_{\mathrm{pb}}\right)}^{2}+{\left(G_{\mathrm{ps}}-G_{\mathrm{pb}}\right)}^{2}+\left(B_{\mathrm{ps}}-B_{\mathrm{pb}}\right)}2,$$

where subscripts *ps* and *pb* represent the proportions for skink and background, respectively. We assumed that lower contrast values indicate higher similarities between body and background (i.e., high background matching).

### 2.6 Statistical analyses

All statistical analyses were conducted using R version 3.2.3 (R Foundation for Statistical Computing).

#### 2.6.1 Color and pattern background matching

First, we tested if background matching was higher for dorsal than ventral body regions. We calculated the differences between the color contrasts against the backgrounds of each three body areas (dorsal, ventral, and ventral tail) using paired Wilcox signed‐rank test. Based on these results, we focused on the body region most “exposed” to predators—the dorsal region.

We then determined if variation in dorsal background matching was influenced by population structure, pattern types, local spatial distance, and season. We used achromatic and chromatic contrasts as our separate response variables: age (juvenile vs. adult), pattern type (*plain*, *midplain*, *spot,* and *midspot*), vegetation gradient (proportion of vegetation cover), and biological seasons as fixed effects; and surveyed years (2006–2008) as a random effect. We categorized seasons based on observations of breeding activity in the field and captivity (M. Baling unpublished data) as (a) nonbreeding (nb, April–September), (b) breeding/mating season (bm, October–December), and (c) breeding/birthing season (bb, January–March). We performed model comparisons using Akaike's information criterion with a correction for finite sample sizes (AIC_c_) to select the linear mixed effects (LME) models (via maximum likelihood) with the lowest AIC_c_ values (R packages lme4 version 1.1‐13and AICcmodavg version 2.1‐1).

To assess color variation independently of background matching, we determined if body colors, pattern types, and habitat background varied with population structure, vegetation gradient, and season. We conducted a stepwise procedure to select the lowest AICc values for LME and general linear mixed effects (GLME) models. We used hue, saturation, and brightness of the dorsal body, ventral body, ventral tail and background as separate response variables; age, pattern type, season, and percentage of vegetation cover as fixed effects; and year of surveys as a random effect for the LME models. We used GLME models (family = binomial) with pattern type as the response variable, sex, season, and percentage of vegetation cover as fixed effects, and year as a random effect for these models.

#### 2.6.2 Seasonal variation in color patterns for intraspecific signaling in adults

In adult shore skinks, we looked for evidence of sex and condition dependence of color patterns between the seasons. We first tested for differences between gravid and nongravid females to rule out color differences due to reproductive status (Cooper Jr., 1983; Cuervo & Belliure, 2013; Ferguson, 1976; Forsman & Shine, 1995; Vercken, Massot, Sinervo, & Clobert, 2008; Weiss, 2006). Because there were no significant differences, we pooled all females in subsequent analyses (ANOVA, hue: *p* = .19, saturation: *p* = .06, brightness: *p* = .65, *n* = 171). We then used hue, saturation, and brightness of dorsal, ventral, and ventral tail as separate response variables; sex, pattern, season, body size (SVL), and body condition (scaled mass index; for methods see Peig & Green, 2009) as fixed effects; and year of surveys as a random effect for the LME models. For pattern types, we used sex, season, body size, and body condition for fixed, and year as random effects for the GLME models.

We examined the fixed variables in all models stated above using likelihood ratio tests to confirm the best model fit and used Tukey's contrast tests (R package multcomp version 1.4.8) *post hoc* to conduct multiple pairwise comparisons of the fixed variables and their interactions. Regressions were used *post hoc* for vegetation cover. Wilcox signed‐rank test was used *post hoc* to detect differences in habitat use (vegetation cover) between the pattern types.

## 3 RESULTS

A total of 352 images of individual skinks and their backgrounds were used in the final analyses. These images were collected in 2006 (*n* = 19), 2007 (*n* = 271) and 2008 (*n* = 62).

### 3.1 Color contrasts among body regions

**Figure 3:**
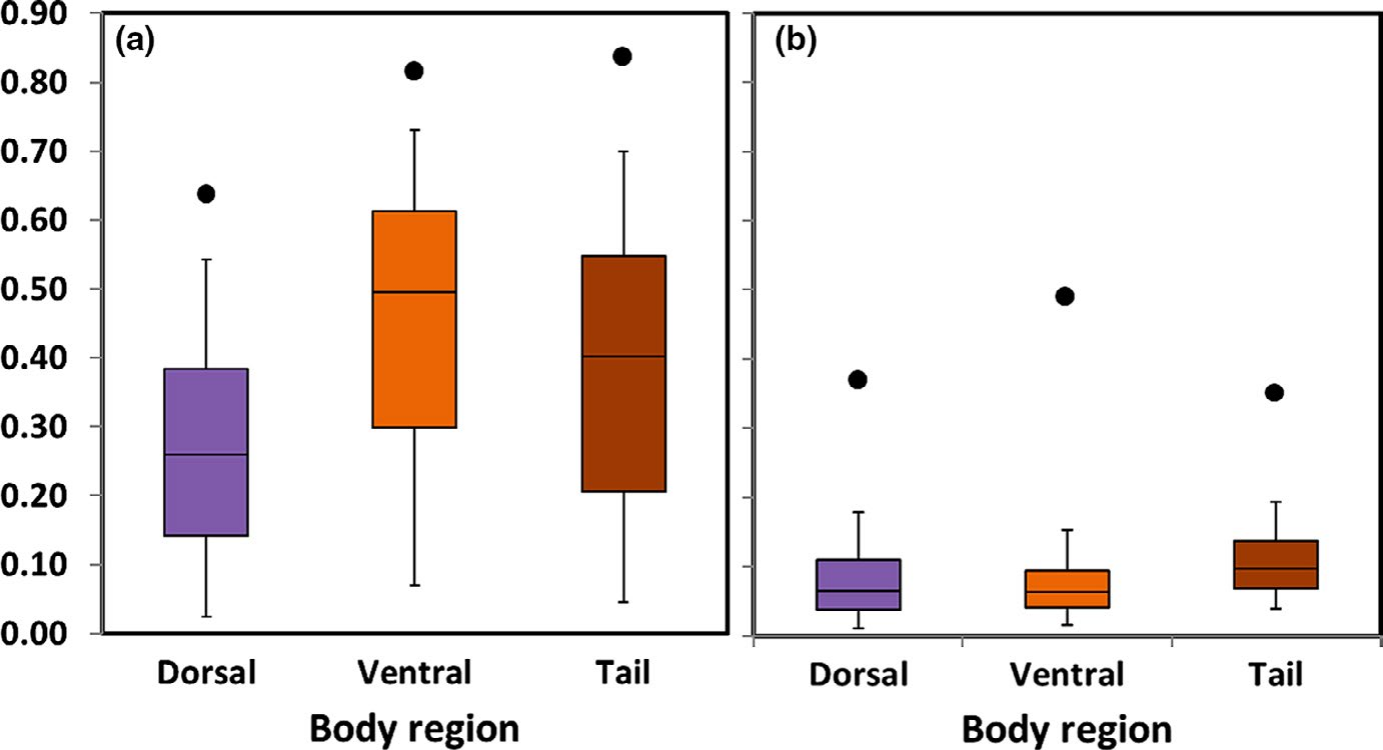
Color contrasts between body regions (dorsal body, ventral body, and ventral tail) and habitat background of shore skinks: a) achromatic contrast and b) chromatic contrast. (*n* = 352)

Overall, shore skinks at Tāwharanui were more camouflaged dorsally than ventrally. The dorsal region had the lowest achromatic contrast compared with the ventral tail and ventral areas (Wilcox test, *p* < .01 for all, *n* = 352; Figure 3). Chromatic contrast values were similar between the dorsal and ventral regions, and both were significantly lower compared with the ventral tail (Wilcox test, dorsal vs. ventral *p* = .45; *p* < .01 for others, *n* = 352).

### 3.2 Color patterns and background matching across the vegetation gradient

**Figure 4:**
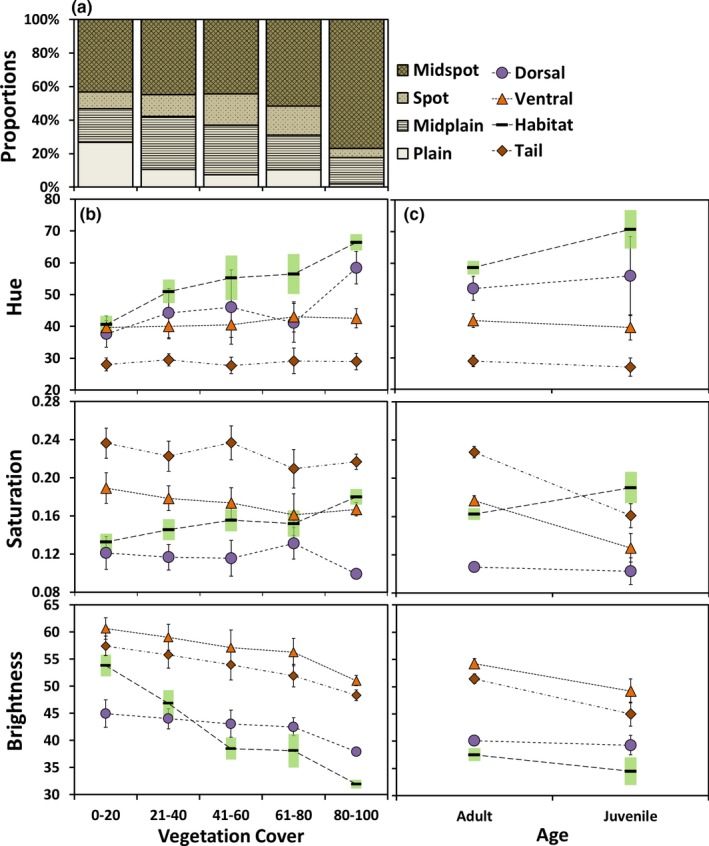
Color pattern trends of shore skinks at Tāwharanui. a) Frequency of four pattern types of skinks across the categorical percentage of vegetation cover. Mean color values (hue, saturation, and brightness) of body regions (dorsal, ventral, and ventral tail) and habitat background across b) the categorical percentage of vegetation cover; and c) age (adult and juvenile) of shore skinks. Bars represent 95% confidence intervals

Skinks were less matched in color (chromatic contrast) as vegetation cover increased, but achromatic contrast remained consistent throughout (linear regression, chromatic contrast: *p* < .01, *r* ^2^ = 0.218; achromatic contrast: *p* = .14, *r* ^2^ = 0.004, Table S1). When we looked at background and skink brightness separately, skinks were on average, darker than their background when vegetation cover was low. However, background brightness steeply declined with increasing vegetation cover, resulting in skinks being lighter than backgrounds with high cover (linear regression, *p* < .01 for both vs. vegetation cover; dorsal: *r* ^2^ = 0.20; background *r* ^2^ = 0.60; Figure 4b; Table S1). Furthermore, the hue and saturation of the background increased (i.e., more yellow‐green) with vegetation cover, resulting in observed higher chromatic contrast between skinks and their backgrounds (linear regression, background: *p* < .01 for both vs. vegetation cover; H: *r* ^2^ = 0.23; S: *r* ^2^ = 0.12; Figure 4b; Table S1). Dorsal saturation values were consistently low (Figure 4b); hence, we described the skinks' dorsal colors as gray‐tones. Dorsal hue value increased with vegetation cover only during the nonbreeding and mating seasons (brown‐gray to green‐gray; linear regression, nb: *p* < .01, *r* ^2^ = 0.16; bm: *p* = .01, *r* ^2^ = 0.06; bb: *p* = .17, *r* ^2^ = 0.01; Table S1).

Shore skink pattern types were spatially structured across the vegetation gradient, where the complexity of dorsal patterning increased across high vegetation cover (Figure 4a; Kruskal–Wallis Test, *p* < .001, *df* = 3, *H* = 40.24). The *plain* type had the lowest abundance within the population (*n* = 21; *spot* *n* = 32, *midplain* *n* = 67, *spot* *n* = 232) and was found more often at locations with low vegetation cover (e.g., 0%–20% vegetation cover = 26.7% vs. 81%–100% vegetation cover = 6.2%; Wilcoxon rank‐sum test, *plain*: *p ≤ *0.01, *W* = 414–959). In contrast, *midspot*, the most complex patterned type (and most abundant in the population), was observed at high vegetation cover (Wilcoxon rank‐sum test; *midspot*: *p* < .01, *W* = 2347–5233). The occurrence of *midplain* and *spot* did not differ with vegetation cover (Wilcoxon rank‐sum test, *p* = .87, *W* = 1,010). Despite the structure, the distributions of pattern types overlapped one another throughout the vegetation gradient.

### 3.3 Association of color to age, sex, and quality

**Figure 5:**
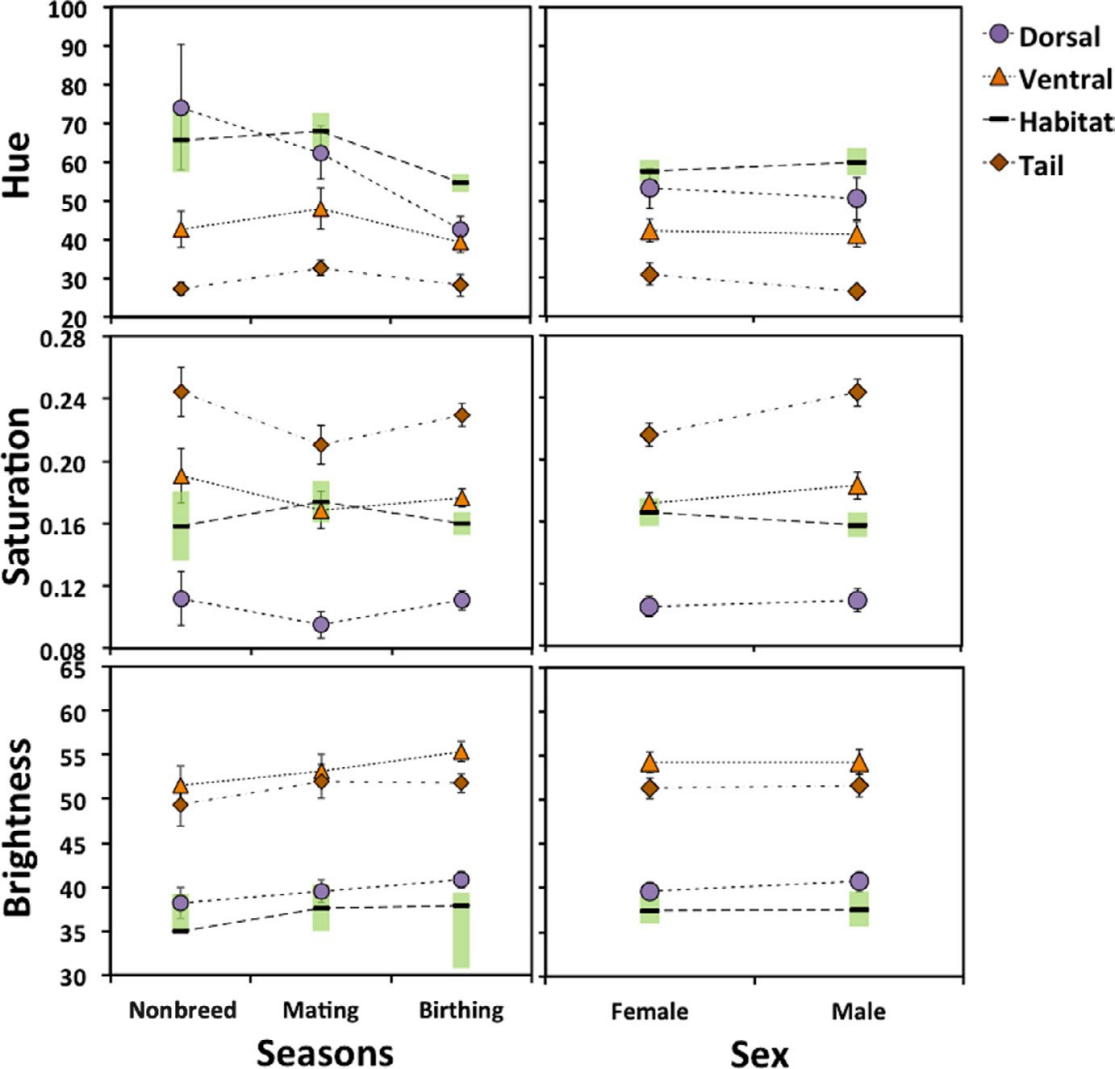
Color pattern trends of adult shore skinks at Tāwharanui. Mean color values (hue, saturation, and brightness) for body regions (dorsal, ventral, and ventral tail) and habitat background across a) seasons and b) sexes. Bars represent 95% confidence intervals

There were differences in color within age classes and sexes for the ventral body regions. Adult shore skinks’ ventral body regions (including tail) were lighter and more intensely orange‐brown than juveniles (Tukey's contrast tests; *p* < .01 for all; Figure 4c; Table S1). Similarly, within adults, ventral regions of males were even more intensely orange‐brown, and tails were more red‐brown compared with females (Tukey's contrast tests, ventral saturation: *p* ≤ 0.01–0.02; ventral tail hue: *p* < .01; Figure 5b; Table S2). There was some evidence of condition dependence of color. Dorsal hue values of adult shore skinks were weakly correlated with body size and body condition, where larger or better‐conditioned skinks were more orange‐gray during the birthing season (linear regression, size: *p* = .03, *r* ^2^ = 0.02; condition: *p* = .04, *r* ^2^ = 0.02; Table S2).

### 3.4 Seasonal variation of color patterns and background matching in adults

Overall, the degree of background matching was not significantly different between seasons for color (Tukey's contrast test, achromatic contrasts between seasons: *p* = .41–.57; chromatic contrast: nb vs. bm *p* = .07) and pattern (Tukey's contrast test, *p* = .74–.99; Table S2). The only exception was for chromatic contrast, where adult shore skinks better matched the background during the birthing season compared with nonbreeding and mating seasons (Tukey's contrast test, *p* < .01; bb = 0.055 ± 0.00003 *SE*; nb = 0.110 ± 0.00007 *SE*; bm = 0.074 ± 0.00004 *SE*; Table S2).

During the birthing season, both males and females had the lowest dorsal hue values, being more brown‐gray compared with other seasons (Tukey's contrast tests, male: *p* < .01 for all comparisons; female: bb vs. nb, *p* < .01; Table S2). In the mating season, all adults were least intense in yellow‐gray and were more yellow‐brown ventrally. During this time, ventral hue value was significantly higher compared with other seasons (Tukey's contrast tests, *p* ≤ .01–.01; Figure 5a; Table S2). During the nonbreeding season, skinks had the most intense green‐gray dorsal coloration and dark orange‐brown ventral coloration. Specifically, saturation values were significantly high for all body regions compared with other seasons (Tukey's contrast tests, dorsal: *p* ≤ .01–.01 ventral: *p* < .01–.05; ventral tail: *p* < .01–.06; Figure 5a; Table S2), while ventral body and ventral tail were darkest (Tukey's contrast test, ventral: nb vs. bb, *p* = .03; ventral tail: nb vs. bm, *p* = .06; Table S2).

## 4 DISCUSSION

Shore skink coloration at the coastal dunes of Tāwharanui showed spatial and temporal variation that is consistent with both crypsis and intraspecific signaling requirements. The structured spatial distribution of the four color pattern types suggests that variants may specialize to the different proportions of vegetation cover. However, the distribution of each four patterns largely overlapped with one another and achromatic contrast of the population was constant across the vegetation gradient, which also suggests that variants may still be well‐camouflaged despite not being in their “optimal” background (e.g., *midspot* present in 0%–20% vegetation cover). In this population, the intensity of body color (i.e., saturation) may not be as important for camouflage as brightness and color patterns. Instead, saturation may be more important for intraspecific signaling, as suggested by the differences in saturation among age classes (adults and juveniles) and sexes on the “hidden” ventral body regions. Adult shore skinks also showed color change between seasons; being dark and rich in color (dorsal green‐gray, ventral orange‐brown) during the nonbreeding season, becoming less intense in color (ventral orange‐brown) during the mating season, to a more brown‐red dorsal color during the birthing season. Hue values in adults were also weakly associated with body size and body condition only during the birthing season. These changes, however, did not significantly affect the degree of background matching over time.

### 4.1 Background matching across a continuous heterogeneous habitat

As predicted within continuous heterogeneous habitats, background matching (at least in terms of achromatic contrast) remained constant across the vegetation gradient. However, how the shore skinks contrasted with their background varied. Overall, skinks were darker than their background at low vegetation cover and were lighter than their background at high vegetation cover. This spatial change in background matching was primarily due to changes in background colors, rather than by variation in skink color per se. It is likely that the trend of constant achromatic contrast was mainly driven by high proportions of dark *midspot* (i.e., the dominant color pattern variant) across the spatial gradient. But whether the survivorship of these dark *midspot*s in suboptimal habitat (e.g., 0%–20% vegetation cover) was achieved by a generalist strategy can only be confirmed by behavioral experiments that examine predation risk of all color patterns on the different backgrounds at the site.

Overall, body patterning of shore skinks matched the vegetation gradient, as expected. Other studies have similarly observed associations of dorsal color pattern types to vegetation types, for example, striped dorsal individuals are found more often in grasses (Chen, Symonds, Melville, & Stuart‐Fox, 2013; Woolbright & Stewart, 2008). Similarly, we noted *midplain* (i.e., striped) individuals in areas of different grasses (e.g., pīngao *Ficinia spiralis*, spinifex *Spinifex sericeus*) at the low to intermediate vegetation cover areas in the foredunes. At higher vegetation cover in the back of the dunes, speckled patterns of the *midspots* complemented the thicker ground vegetation cover such as rushes and tangled, dense vines (e.g., sand coprosma *Coprosma acerosa*, pohuehue *Muehlenbeckia complexa*; Wedding, 2007).

Although this population showed a structured distribution in color pattern matching, it is still unclear why particular color patterns such as *midspot* were found at high frequency when vegetation cover was low (cf. *plain*), where we would expect color pattern matching to be low for this type. We currently do not know the home ranges of these skinks, but the wide distribution of the color pattern variants and low recapture frequency per survey (c. <2% each year) indicate a potentially more considerable degree of mobility of individuals across the vegetation gradient than expected.

Skinks may also have alternative antipredator strategies (e.g., disruptive coloration, flicker fusion; Cooper Jr. & Greenberg, 1992; Endler, 1978; Halperin, Carmel, & Hawlena, 2017; Hogan, Scott‐Samuel, & Cuthill, 2016; Merilaita & Lind, 2005; Stevens & Merilaita, 2011) or other biological functions to color such as thermoregulation (e.g., thermal melanism; Clusella‐Trullas, Wyk, & Spotila, 2007; Stuart‐Fox & Moussalli, 2009) that can affect their distribution.

### 4.2 Color patterns for intraspecific signaling

Shore skinks showed evidence of color differences with respect to age (juveniles vs. adults), sex, and quality. Individuals advertising their maturity and sex through body colors can reduce conspecific aggression, determine social hierarchy, identify competitors, determine the condition or quality of potential mates, and indicate reproductive status (Cuervo & Belliure, 2013; Cuervo & Shine, 2007; Forsman & Shine, 1995; Martin, Meylan, Gomez, & Galliard, 2013; Vercken et al., 2008). There are anecdotal observations of captive wild‐born adult shore skinks that have died due to conspecific aggression (including among females; M. Baling unpublished data), but it is unknown whether the variation in coloration covaries with these behaviors in shore skinks.

### 4.3 Variation of color patterns across seasons

As we expected, shore skinks showed minor color change between nonbreeding and breeding seasons. The largest variance was observed in dorsal hue, changing from green‐gray during the nonbreeding season to yellow‐gray in the mating season to brown‐gray during the birthing season. Contrary to our expectations, however, the degree of background matching was relatively consistent across the vegetation gradient over the seasons. Furthermore, seasonal change in ventral coloration was subtle. Although coloration at the hidden body regions is often argued to function in mate attraction (Cuervo & Shine, 2007; Martin et al., 2013), shore skinks have not been observed to display their ventral side to conspecifics (Meyers, Irschick, Vanhooydonck, & Herrel, 2006; Whiting et al., 2006). Overall, the subtle changes in color, and whether they are conspicuous enough to be perceived by conspecifics or predators requires further investigation.

In conclusion, our study showed how crypsis could play an influential role in generating fine‐scale variations in dorsal coloration of shore skinks in spatially heterogeneous habitats over time. The population exhibited mixed strategies for background matching, where there was some degree of specialization along the vegetation gradient (specialist strategy), but there was significant overlap in the distribution of each color pattern type (generalist strategy). Although the degree of background matching did not vary greatly between seasons, particular aspects of color (e.g., saturation) and the colors of the ventral body region (i.e., not exposed to predators) can still deviate from the expectations of crypsis that would otherwise confer protection.

## CONFLICT OF INTEREST

We have no competing interests.

## AUTHORS' CONTRIBUTIONS

MB obtained permits, planned the study design, collected field data, contributed to statistical analyses, and wrote the manuscript. DSF contributed to statistical analyses and edited the manuscript. DHB contributed to study design, obtained permits, and edited the manuscript. JD contributed to statistical analyses and editing of the manuscript.

## Data Availability

Dryad https://doi.org/10.5061/dryad.h9w0vt4dt.

## References

[ece36024-bib-0001] Baling, M. (2017). Functional significance of highly variable colouration in the shore skink (*Oligosoma smithi*). PhD Thesis. (145 pp.). Auckland, New Zealand: Massey University.

[ece36024-bib-0002] Baling, M. , Stuart‐Fox, D. , Brunton, D. H. , & Dale, J. (2016). Habitat suitability for conservation translocation: The importance of considering camouflage in cryptic species. Biological Conservation, 203, 298–305. 10.1016/j.biocon.2016.10.002

[ece36024-bib-0003] Bond, A. B. (2007). The evolution of color polymorphism: Crypticity, searching images, and apostatic selection. Annual Review of Ecology, Evolution, and Systematics, 8, 489–514. 10.1146/annurev.ecolsys.38.091206.095728

[ece36024-bib-0004] Cadena, V. , Smith, K. R. , Endler, J. A. , & Stuart‐Fox, D. (2017). Geographic divergence and colour change in response to visual backgrounds and illumination intensity in bearded dragons. Journal of Experimental Biology, 220, 1048–1055. 10.1242/jeb.148544 28298465

[ece36024-bib-0005] Caro, T. , Sherratt, T. N. , & Stevens, M. (2016). The ecology of multiple colour defences. Evolutionary Ecology, 30(5), 797–809. 10.1007/s10682-016-9854-3

[ece36024-bib-0006] Chen, I.‐P. , Symonds, M. R. E. , Melville, J. , & Stuart‐Fox, D. (2013). Factors shaping the evolution of colour patterns in Australian agamid lizards (Agamidae): A comparative study. Biological Journal of Linnean Society, 109(1), 101–112. 10.1111/bij.12030

[ece36024-bib-0007] Clusella‐Trullas, S. , Van Wyk, J. H. , & Spotila, J. R. (2007). Thermal melanism in ectotherms. Journal of Thermal Biology, 32, 235–245. 10.1016/j.jtherbio.2007.01.013

[ece36024-bib-0008] Cooper Jr, W. E. (1983). Female color change in the keeled earless lizard, *Holbrookia propinqua*: Relationship to reproductive cycle. The Southwestern Naturalist, 28(3), 275–280.

[ece36024-bib-0009] Cooper Jr, W. E. , & Greenberg, N. (1992). Reptilian coloration and behaviour In Gans C., & Crews D. (Eds.), Biology of the Reptilia: Hormones, brain and behavior. Chicago, IL: The University of Chicago Press.

[ece36024-bib-0010] Cuervo, J. J. , & Belliure, J. (2013). Exploring the function of red colouration in female spiny‐footed lizards (*Acanthodactylus erythrurus*): Patterns of seasonal colour change. Amphibia‐Reptilia, 34, 525–538. 10.1163/15685381-00002912

[ece36024-bib-0011] Cuervo, J. J. , & Shine, R. (2007). Hues of a dragon's belly: Morphological correlates of ventral coloration in water dragons. Journal of Zoology, 273(3), 298–304. 10.1111/j.1469-7998.2007.00328.x

[ece36024-bib-0012] Dale, J. (2006). Intraspecific variation in coloration In Hill G. E., & McGraw K. J. (Eds.), Bird coloration: Function and evolution. Cambridge, MA: Harvard University Press.

[ece36024-bib-0013] Dale, J. , Dey, C. J. , Delhey, K. , Kempenaers, B. , & Valcu, M. (2015). The effects of life history and sexual selection on male and female plumage colouration. Nature, 527, 367–370. 10.1038/nature15509 26536112

[ece36024-bib-0014] Endler, J. A. (1978). A predator's view of animal color patterns. Evolutionary Biology, 11, 319–364. 10.1007/978-1-4615-6956-5_5

[ece36024-bib-0015] Endler, J. A. (1990). On the measurement and classification of colour in studies of animal colour patterns. Biological Journal of Linnean Society, 41(4), 315–352. 10.1111/j.1095-8312.1990.tb00839.x

[ece36024-bib-0016] Ferguson, G. W. (1976). Color change and reproductive cycling in female collared lizards (*Crotaphytus collaris*). Copeia, 3, 491–494. 10.2307/1443364

[ece36024-bib-0017] Forsman, A. , & Shine, R. (1995). The adaptive significance of colour pattern polymorphism in the Australian scincid lizard *Lampropholis delicata* . Biological Journal of the Linnean Society, 55(4), 273–291. 10.1111/j.1095-8312.1995.tb01066.x

[ece36024-bib-0018] Halperin, T. , Carmel, L. , & Hawlena, D. (2017). Movement correlates of lizards’ dorsal pigmentation patterns. Functional Ecology, 31(2), 370–376. 10.1111/1365-2435.12700

[ece36024-bib-0019] Hardy, G. S. (1977). The New Zealand Scincidae (Reptilia: Lacertilia); a taxanomic and zoogeographic study. New Zealand Journal of Zoology, 4(3), 221–325. 10.1080/03014223.1977.9517956

[ece36024-bib-0020] Hitchmough, R. , Barr, B. , Lettink, M. , Monks, J. , Reardon, J. , Tocher, M. , … Rolfe, J. (2016). Conservation status of New Zealand reptiles, 2015 (18 9pp.). Wellington, New Zealand: Department of Conservation.

[ece36024-bib-0021] Hogan, B. G. , Scott‐Samuel, N. E. , & Cuthill, I. C. (2016). Contrast, contours and the confusion effect in dazzle camouflage. Royal Society Open Science, 10.1098/rsos.160180 PMC4968467 27493775

[ece36024-bib-0022] Houston, A. I. , Stevens, M. , & Cuthill, I. C. (2007). Animal camouflage: Compromise or specialize in a 2 patch‐type environment? Behavioral Ecology, 18(4), 769–775. 10.1093/beheco/arm039

[ece36024-bib-0023] Hughes, A. , Liggins, E. , & Stevens, M. (2019). Imperfect camouflage: How to hide in a variable world? Proceedings of the Royal Society B: Biological Sciences, 286(1902), 20190646 10.1098/rspb.2019.0646 PMC6532520 31088268

[ece36024-bib-0024] Maitland, M. (2011). Tawharanui Open Sanctuary ‐ detection and removal of pest incursions In Veitch C. R., Clout M. N., & Towns D. R. (Eds.), Island invasives: Eradication and management (pp. 441–444). Gland, Switzerland: IUCN.

[ece36024-bib-0025] Martin, M. , Meylan, S. , Gomez, D. , & Le Galliard, J.‐F. (2013). Ultraviolet and carotenoid‐based coloration in the viviparous lizard *Zootoca vivipara* (Squamata: Lacertidae) in relation to age, sex, and morphology. Biological Journal of the Linnean Society, 110(1), 128–141. 10.1111/bij.12104

[ece36024-bib-0026] Merilaita, S. (2003). Visual background complexity facilitates the evolution of camouflage. Evolution, 57(6), 1248–1254. 10.1554/03-011 12894933

[ece36024-bib-0027] Merilaita, S. , & Dimitrova, M. (2014). Accuracy of background matching and prey detection: Predation by blue tits indicates intense selection for highly matching prey colour pattern. Functional Ecology, 28, 1208–1215. 10.1111/1365-2435.12248

[ece36024-bib-0028] Merilaita, S. , & Lind, J. (2005). Background‐matching and disruptive coloration, and the evolution of cryptic coloration. Proceedings of the Royal Society B 272, 665–670. 10.1098/rspb.2004.3000 15817442 PMC1564081

[ece36024-bib-0029] Merilaita, S. , Lyytinen, A. , & Mappes, J. (2001). Selection for cryptic coloration in a visually heterogeneous habitat. Proceedings of the Royal Society B, 268, 1925–1929. 10.1098/rspb.2001.1747 11564349 PMC1088829

[ece36024-bib-0030] Merilaita, S. , Tuomi, J. , & Jormalainen, V. (1999). Optimization of cryptic coloration in heterogeneous habitats. Biological Journal of the Linnean Society, 67, 151–161. 10.1111/j.1095-8312.1999.tb01858.x

[ece36024-bib-0031] Meyers, J. J. , Irschick, D. J. , Vanhooydonck, B. , & Herrel, A. (2006). Divergent roles for multiple sexual signals in a polygynous lizard. Functional Ecology, 20(4), 709–716. 10.1111/j.1365-2435.2006.01152.x

[ece36024-bib-0032] Mills, L. S. , Zimova, M. , Oyler, J. , Running, S. , Abatzoglou, J. T. , & Lukacs, P. M. (2013). Camouflage mismatch in seasonal coat color due to decreased snow duration. Proceedings of the National Academy of Sciences, 110(18), 7360–7365. 10.1073/pnas.1222724110 PMC3645584 23589881

[ece36024-bib-0033] Montgomerie, R. (2006). Analyzing colors In Hill G. E., & McGraw K. J. (Eds.), Bird coloration: Mechanisms and measurements (pp. 90–147). Cambridge, MA: Harvard University Press.

[ece36024-bib-0034] Nilsson, J. , & Ripa, J. (2010). The origin of polymorphic crypsis in a heterogenous environment. Evolution, 64(5), 1386–1394. 10.1111/j.1558-5646.2009.00918.x 20002166

[ece36024-bib-0035] Peig, J. , & Green, J. (2009). New perspectives for estimating body condition from mass/length data: The scaled mass index as an alternative method. Oikos, 118(12), 1883–1891. 10.1111/j.1600-0706.2009.17643.x

[ece36024-bib-0036] Rojas, B. (2016). Behavioural, ecological, and evolutionary aspects of diversity in frog colour patterns. Biological Reviews, 10.1111/brv.12269 27020467

[ece36024-bib-0037] Smith, K. R. , Cadena, V. , Endler, J. A. , Kearney, M. R. , Porter, W. P. , & Stuart‐Fox, D. (2016). Color change for thermoregulation versus camouflage in free‐ranging lizards. The American Naturalist, 188(6), 668–678. 10.1086/688765 27860512

[ece36024-bib-0038] Steen, J. B. , Erikstad, K. E. , & Høidal, K. (1992). Cryptic behaviour in moulted hen willow *Ptarmigan lagopus l. lagopus* during snow melt. Ornis Scandinavica, 23(1), 101–104.

[ece36024-bib-0039] Stevens, M. , & Merilaita, S. (2011). Animal camouflage: An introduction In Stevens M., & Merilaita S. (Eds.), Animal camouflage: Mechanisms and function (pp. 1–16). Cambridge, UK: Cambridge University Press.

[ece36024-bib-0040] Stevens, M. , Párraga, C. A. , Cuthill, I. C. , Partridge, J. C. , & Troscianko, T. S. (2007). Using digital photography to study animal coloration. Biological Journal of Linnean Society, 90(2), 211–237. 10.1111/j.1095-8312.2007.00725.x

[ece36024-bib-0041] Stuart‐Fox, D. , & Moussalli, A. (2009). Camouflage, communication and thermoregulation: Lessons from colour changing organisms. Philosophical Transactions of the Royal Society of London B, 364, 463–470. 10.1098/rstb.2008.0254 PMC2674084 19000973

[ece36024-bib-0042] Stuart‐Fox, D. M. , Moussalli, A. , Johnston, G. R. , & Owens, I. P. F. (2004). Evolution of color variation in dragon lizards: Quantitative tests of the role of crypsis and local adaptation. Evolution, 58(7), 1549–1559. 10.1554/03-448 15341157

[ece36024-bib-0043] Stuart‐Fox, D. M. , & Ord, T. J. (2004). Sexual selection, natural selection and the evolution of dimorphic coloration and ornamentation in agamid lizards. Proceedings of the Royal Society B 271, 2249–2255. 10.1098/rspb.2004.2802 15539350 PMC1691857

[ece36024-bib-0044] Towns, D. R. (1975). Ecology of the black shore skink, *Leiolopisma suteri* (Lacertilia: Scincidae), in boulder beach habitats. New Zealand Journal of Ecology, 2(4), 389–407.

[ece36024-bib-0045] Towns, D. R. , Neilson, K. A. , & Whitaker, A. H. (2002). North Island *Oligosoma* spp. Skink Recovery Plan (62 pp.). Wellington, New Zealand: Department of Conservation.

[ece36024-bib-0046] Tullberg, B. S. , Gamberale‐Stille, G. , Bohlin, T. , & Merilaita, S. (2008). Seasonal ontogenetic colour plasticity in the adult striated shieldbug *Graphosoma lineatum* (Heteroptera) and its effect on detectability. Behavioral Ecology and Sociobiology, 62(9), 1389–1396. 10.1007/s00265-008-0567-7

[ece36024-bib-0047] Valcu, M. , & Dale, J. (2014). colorZapper: color extraction utilities. R package version 1.0.https://github.com/valcu/colorZapper

[ece36024-bib-0048] Vercken, E. , Massot, M. , Sinervo, B. , & Clobert, J. (2008). Colour variation and alternative reproductive strategies in females of the common lizard *Lacerta vivipara* . Journal of Evolutionary Biology, 20(1), 221–232. 10.1111/j.1420-9101.2006.01208.x 17210015

[ece36024-bib-0049] Wedding, C. J. (2007). Aspects of the impacts of mouse (*Mus musculus*) control on skinks in Auckland (p. 146). Msc. Auckland, New Zealand: Massey University.

[ece36024-bib-0050] Weiss, S. L. (2006). Female‐specific color is a signal of quality in the striped plateau lizard (*Sceloporus virgatus*). Behavioral Ecology, 17(5), 726–732. 10.1093/beheco/arl001

[ece36024-bib-0051] Whiting, M. J. , Stuart‐Fox, D. , O'Connor, D. , Firth, D. , Bennett, N. C. , & Blomberg, S. P. (2006). Ultraviolet signals ultra‐aggression in a lizard. Animal Behaviour, 72, 353–363. 10.1016/j.anbehav.2005.10.018

[ece36024-bib-0052] Woolbright, L. L. , & Stewart, M. M. (2008). Spatial and temporal variation in color pattern morphology in the tropical frog, *Eleutherodactylus coqui* . Copeia, 2008(2), 431–437. 10.1643/CG-06-092

[ece36024-bib-0053] Zimova, M. , Mills, L. S. , Lukacs, P. M. , & Mitchell, M. S. (2014). Snowshoe hares display limited phenotypic plasticity to mismatch in seasonal camouflage. Proceedings of the Royal Society B, 281(1782), 20140029 10.1098/rspb.2014.0029 24619446 PMC3973274

